# Supplementary material for: Incidence and prevalence of obstructive sleep apnoea and narcolepsy in the UK: a population-based descriptive study
Source: Thorax. 2026 Feb 5;81(9):e223863. doi: 10.1136/thorax-2025-223863 (PMC13479645; doi:10.1136/thorax-2025-223863)
Supplement: Supplementary file 1 [file thorax-81-9-s001.docx]

**SUPPLEMENTARY APPENDIX**

**UK incidence and prevalence of Obstructive Sleep Apnoea and narcolepsy: a population-based descriptive study**

**SA Figure 1:**

Title: Incidence rate by months since registration in CPRD practice

Caption: OSA = Obstructive Sleep Apnoea. Black dotted line = 3 months since registration

**SA Figure 2: Incidence Rate Ratios by calendar year adjusted for age, sex and BMI (OSA only)**

**SA Figure 3:** Age, sex and BMI adjusted incident Rate Ratios by demographic characteristics for diagnosed OSA in adults in England from 2000-2019, stratified by 5-year time period.

**SA Figure 4:** Age and sex adjusted Incident Rate Ratios by demographic characteristics for diagnosed narcolepsy in England from 2000-2019, stratified by 5-year time period.

**SA Figure 5:** Crude incidence and standardised prevalence of OSA and narcolepsy over time in linked and primary care only data.

**SA Figure 6:** Crude incidence and standardised prevalence of OSA and narcolepsy over time in each UK nation (primary care only data).

Caption: Prevalence rates are standardized to the age-sex distribution of each country in each year

**SA Table 1**: **Data sources, versions and DOIs**

| **Database** | **Version** | **DOI** |
| --- | --- | --- |
| CPRD Aurum | 2022.03.001 | https://doi.org/10.48329/MY9S-4X08 |
| CPRD GOLD | 2022.03.001 | https://doi.org/10.48329/5RHX-ER29 |
| CPRD GOLD HES APC | 2022.01.001 | https://doi.org/10.48329/fagm-ez75 |
| CPRD Aurum HES APC | 2022.01.001 | https://doi.org/10.48329/vagx-9d96 |
| CPRD GOLD ONS deaths | 2022.01.001 | https://doi.org/10.48329/wq7v-x832 |
| CPRD Aurum ONS deaths | 2022.01.001 | https://doi.org/10.48329/Q34F-F505 |
| CPRD GOLD Small Area data (practice) – Carstairs Index | 2022.01.001 | https://doi.org/10.48329/aep7-aq95 |
| CPRD Aurum Small Area data (practice) – Carstairs Index | 2022.01.001 | https://doi.org/10.48329/3saw-rz63 |
| CPRD GOLD Small Area data (patient) – rural-urban classification | 2022.01.001 | https://doi.org/10.48329/y101-0w14 |
| CPRD Aurum Small Area data (patient) – rural-urban classification | 2022.01.001 | https://doi.org/10.48329/aytt-h222 |

**SA Table 2:** Composition of prevalent and incident cases and denominators

A: Obstructive Sleep Apnoea (OSA) in adults (2019)

B: Narcolepsy in the full population (2019 prevalence, 2014 to 2019 incidence)

| **A (OSA)** | **Prevalent cases** | **Study population (n)** | **Incident cases** | **Person-years (/100 000)** |
| --- | --- | --- | --- | --- |
| **Full study population** | 143277 (100.0%) | 10360607 (100.0%) | 17515 (100.0%) | 102.4596 (100.0%) |
| Age group |  |  |  |  |
| 18 to < 25 | 694 (0.5%) | 1023006 (9.9%) | 328 (1.9%) | 10.74129 (10.5%) |
| 25 to < 35 | 5737 (4.0%) | 1936833 (18.7%) | 1470 (8.4%) | 19.23007 (18.8%) |
| 35 to < 45 | 15688 (10.9%) | 1821663 (17.6%) | 2756 (15.7%) | 18.00595 (17.6%) |
| 45 to < 55 | 32549 (22.7%) | 1800111 (17.4%) | 4225 (24.1%) | 17.64058 (17.2%) |
| 55 to < 65 | 40265 (28.1%) | 1548317 (14.9%) | 4331 (24.7%) | 15.05144 (14.7%) |
| 65 to < 75 | 31837 (22.2%) | 1182036 (11.4%) | 2880 (16.4%) | 11.48233 (11.2%) |
| 75 to < 85 | 13910 (9.7%) | 734407 (7.1%) | 1291 (7.4%) | 7.194917 (7.0%) |
| 85 plus | 2597 (1.8%) | 314234 (3.0%) | 234 (1.3%) | 3.113063 (3.0%) |
| **Sex** |  |  |  |  |
| male | 101049 (70.5%) | 5176054 (50.0%) | 11232 (64.1%) | 50.92628 (49.7%) |
| female | 42228 (29.5%) | 5184553 (50.0%) | 6283 (35.9%) | 51.53336 (50.3%) |
| **Ethnicity** |  |  |  |  |
| 0. White | 123610 (86.3%) | 7929876 (76.5%) | 14786 (84.4%) | 78.22609 (76.3%) |
| 1. South Asian | 8162 (5.7%) | 730483 (7.1%) | 1166 (6.7%) | 7.262222 (7.1%) |
| 2. Black | 5753 (4.0%) | 421808 (4.1%) | 703 (4.0%) | 4.192815 (4.1%) |
| 3. Other | 2158 (1.5%) | 304707 (2.9%) | 308 (1.8%) | 3.032032 (3.0%) |
| 4. Mixed | 1440 (1.0%) | 142490 (1.4%) | 192 (1.1%) | 1.422215 (1.4%) |
| Missing | 2154 (1.5%) | 831243 (8.0%) | 360 (2.1%) | 8.324272 (8.1%) |
| **Region (number of practices)** |  |  |  |  |
| North East (52) | 5655 (3.9%) | 318806 (3.1%) | 543 (3.1%) | 3.190468 (3.1%) |
| North West (309) | 24223 (16.9%) | 1913401 (18.5%) | 3216 (18.4%) | 18.94195 (18.5%) |
| Yorks & The Humber (41) | 4955 (3.5%) | 360693 (3.5%) | 566 (3.2%) | 3.57356 (3.5%) |
| East Midlands (35) | 3044 (2.1%) | 242177 (2.3%) | 334 (1.9%) | 2.397704 (2.3%) |
| West Midlands (277) | 24010 (16.8%) | 1643109 (15.9%) | 3006 (17.2%) | 16.23146 (15.8%) |
| East of England (44) | 6508 (4.5%) | 413357 (4.0%) | 591 (3.4%) | 4.072668 (4.0%) |
| London (318) | 25672 (17.9%) | 2160670 (20.9%) | 3225 (18.4%) | 21.44683 (20.9%) |
| South East (239) | 30567 (21.3%) | 2076796 (20.0%) | 3817 (21.8%) | 20.60352 (20.1%) |
| South West (145) | 18643 (13.0%) | 1231598 (11.9%) | 2217 (12.7%) | 12.00149 (11.7%) |
| **Area based deprivation** |  |  |  |  |
| 1 (least deprived) | 18673 (13.0%) | 1299876 (12.5%) | 2144 (12.2%) | 12.88049 (12.6%) |
| 2 | 27961 (19.5%) | 2067191 (20.0%) | 3372 (19.3%) | 20.54984 (20.1%) |
| 3 | 32279 (22.5%) | 2348653 (22.7%) | 3851 (22.0%) | 23.1538 (22.6%) |
| 4 | 31323 (21.9%) | 2166843 (20.9%) | 3884 (22.2%) | 21.32207 (20.8%) |
| 5 (most deprived) | 33041 (23.1%) | 2478044 (23.9%) | 4264 (24.3%) | 24.55345 (24.0%) |
| **Urban-rural** |  |  |  |  |
| urban | 119129 (83.1%) | 8778308 (84.7%) | 14908 (85.1%) | 86.88827 (84.8%) |
| rural | 24034 (16.8%) | 1572456 (15.2%) | 2593 (14.8%) | 15.47453 (15.1%) |
| Missing | 114 (0.1%) | 9843 (0.1%) | 14 (0.1%) | .0968552 (0.1%) |
| **Practice size quintile** |  |  |  |  |
| 1 largest | 50437 (35.2%) | 3941366 (38.0%) | 6225 (35.5%) | 38.99069 (38.1%) |
| 2 | 35019 (24.4%) | 2411357 (23.3%) | 4234 (24.2%) | 23.82257 (23.3%) |
| 3 | 26774 (18.7%) | 1859351 (17.9%) | 3242 (18.5%) | 18.41168 (18.0%) |
| 4 | 19693 (13.7%) | 1341764 (13.0%) | 2462 (14.1%) | 13.21102 (12.9%) |
| 5 smallest | 11354 (7.9%) | 806769 (7.8%) | 1352 (7.7%) | 8.023686 (7.8%) |
| **BMI category** |  |  |  |  |
| Underweight | 432 (0.3%) | 235733 (2.3%) | . (.%) | . (.%) |
| Normal weight | 11986 (8.4%) | 3508945 (33.9%) | 1682 (9.6%) | 34.9553 (34.1%) |
| Overweight | 33904 (23.7%) | 2995206 (28.9%) | 3959 (22.6%) | 29.38917 (28.7%) |
| Obesity class I | 38244 (26.7%) | 1401261 (13.5%) | 4275 (24.4%) | 13.45145 (13.1%) |
| Obesity class II | 26695 (18.6%) | 520964 (5.0%) | 3172 (18.1%) | 4.843572 (4.7%) |
| Obesity class III+ | 29091 (20.3%) | 289112 (2.8%) | 3645 (20.8%) | 2.51635 (2.5%) |
| Missing | 2925 (2.0%) | 1409386 (13.6%) | 782 (4.5%) | 17.30381 (16.9%) |

| **B (narcolepsy)** | **Prevalent cases** | **Study population (n)** | **Incident cases** | **Person-years (/100 000)** |
| --- | --- | --- | --- | --- |
| **Full study population** | 2662 (100.0%) | 13159742 (100.0%) | 761 (100.0%) | 645.9674 (100.0%) |
| **Age group** |  |  |  |  |
| 0 to < 9 | 21 (0.8%) | 1270179 (9.7%) | 30 (3.9%) | 64.74413 (10.0%) |
| 9 to < 18 | 108 (4.1%) | 1386937 (10.5%) | 88 (11.6%) | 65.72792 (10.2%) |
| 18 to < 25 | 168 (6.3%) | 1161086 (8.8%) | 94 (12.4%) | 56.1519 (8.7%) |
| 25 to < 35 | 399 (15.0%) | 1939578 (14.7%) | 146 (19.2%) | 94.121 (14.6%) |
| 35 to < 45 | 509 (19.1%) | 1822046 (13.8%) | 133 (17.5%) | 89.08833 (13.8%) |
| 45 to < 55 | 488 (18.3%) | 1800325 (13.7%) | 106 (13.9%) | 91.5315 (14.2%) |
| 55 to < 65 | 364 (13.7%) | 1548575 (11.8%) | 69 (9.1%) | 74.1439 (11.5%) |
| 65 to < 75 | 277 (10.4%) | 1182240 (9.0%) | 43 (5.7%) | 59.23645 (9.2%) |
| 75 to < 85 | 220 (8.3%) | 734519 (5.6%) | 33 (4.3%) | 35.62558 (5.5%) |
| 85 plus | 108 (4.1%) | 314257 (2.4%) | 19 (2.5%) | 15.59666 (2.4%) |
| **Sex** |  |  |  |  |
| male | 1245 (46.8%) | 6611703 (50.2%) | 337 (44.3%) | 324.2886 (50.2%) |
| female | 1417 (53.2%) | 6548039 (49.8%) | 424 (55.7%) | 321.6787 (49.8%) |
| **Ethnicity** |  |  |  |  |
| 0. White | 2261 (84.9%) | 9939848 (75.5%) | 621 (81.6%) | 495.1721 (76.7%) |
| 1. South Asian | 77 (2.9%) | 1011101 (7.7%) | 31 (4.1%) | 47.69589 (7.4%) |
| 2. Black | 186 (7.0%) | 585159 (4.4%) | 62 (8.1%) | 28.18765 (4.4%) |
| 3. Other | 28 (1.1%) | 391117 (3.0%) | 14 (1.8%) | 17.34145 (2.7%) |
| 4. Mixed | 53 (2.0%) | 255972 (1.9%) | 21 (2.8%) | 11.62666 (1.8%) |
| Missing | 57 (2.1%) | 976545 (7.4%) | 12 (1.6%) | 45.94366 (7.1%) |
| **Region** |  |  |  |  |
| North East (52) | 91 (3.4%) | 394775 (3.0%) | 17 (2.2%) | 20.4515 (3.2%) |
| North West (309) | 455 (17.1%) | 2421981 (18.4%) | 136 (17.9%) | 117.2262 (18.1%) |
| Yorks & The Humber (41) | 72 (2.7%) | 445481 (3.4%) | 14 (1.8%) | 22.89745 (3.5%) |
| East Midlands (35) | 58 (2.2%) | 295175 (2.2%) | 19 (2.5%) | 15.44438 (2.4%) |
| West Midlands (277) | 431 (16.2%) | 2095827 (15.9%) | 153 (20.1%) | 103.0279 (15.9%) |
| East of England (44) | 105 (3.9%) | 533414 (4.1%) | 20 (2.6%) | 28.51435 (4.4%) |
| London (318) | 581 (21.8%) | 2773362 (21.1%) | 168 (22.1%) | 130.9392 (20.3%) |
| South East (239) | 560 (21.0%) | 2653194 (20.2%) | 157 (20.6%) | 130.6536 (20.2%) |
| South West (145) | 309 (11.6%) | 1546533 (11.8%) | 77 (10.1%) | 76.8128 (11.9%) |
| **Area based deprivation** |  |  |  |  |
| 1 (least deprived) | 325 (12.2%) | 1658427 (12.6%) | 91 (12.0%) | 80.49815 (12.5%) |
| 2 | 507 (19.0%) | 2588319 (19.7%) | 119 (15.6%) | 127.9654 (19.8%) |
| 3 | 613 (23.0%) | 2945071 (22.4%) | 184 (24.2%) | 145.3312 (22.5%) |
| 4 | 604 (22.7%) | 2747216 (20.9%) | 183 (24.0%) | 134.9633 (20.9%) |
| 5 (most deprived) | 613 (23.0%) | 3220709 (24.5%) | 184 (24.2%) | 157.2094 (24.3%) |
| **Urban-rural** |  |  |  |  |
| urban | 2279 (85.6%) | 1.12e+07 (85.0%) | 667 (87.6%) | 547.0087 (84.7%) |
| rural | 381 (14.3%) | 1959232 (14.9%) | 94 (12.4%) | 98.34284 (15.2%) |
| Missing | 2 (0.1%) | 12076 (0.1%) | 0 (0.0%) | .6158447 (0.1%) |
| **Practice size quintile** |  |  |  |  |
| 1 largest | 989 (37.2%) | 4953260 (37.6%) | 280 (36.8%) | 238.9982 (37.0%) |
| 2 | 669 (25.1%) | 3072493 (23.3%) | 188 (24.7%) | 152.2185 (23.6%) |
| 3 | 474 (17.8%) | 2360250 (17.9%) | 132 (17.3%) | 118.3416 (18.3%) |
| 4 | 332 (12.5%) | 1731268 (13.2%) | 106 (13.9%) | 85.51196 (13.2%) |
| 5 smallest | 198 (7.4%) | 1042471 (7.9%) | 55 (7.2%) | 50.89715 (7.9%) |

**SA Table 3:** Diagnosed incidence rates for Obstructive Sleep Apneoa (OSA) and narcolepsy in England from 2000 to 2019

| **Year** | **Incident cases** | **Person years / 100 000** | **Crude incidence rate per 100 000 person years (95% CI)** |
| --- | --- | --- | --- |
| **Obstructive Sleep Apnoea (adults only)** | | | |
| 2000 | 2546 | 86.566 | 29.411 (28.291-30.576) |
| 2001 | 2933 | 88.505 | 33.139 (31.961-34.361) |
| 2002 | 3486 | 90.369 | 38.575 (37.316-39.877) |
| 2003 | 3775 | 92.275 | 40.910 (39.626-42.236) |
| 2004 | 4086 | 93.947 | 43.492 (42.179-44.847) |
| 2005 | 4486 | 95.092 | 47.175 (45.815-48.576) |
| 2006 | 5184 | 96.574 | 53.679 (52.238-55.161) |
| 2007 | 6022 | 98.119 | 61.375 (59.844-62.944) |
| 2008 | 7843 | 99.518 | 78.810 (77.085-80.574) |
| 2009 | 8457 | 100.165 | 84.431 (82.650-86.250) |
| 2010 | 9064 | 100.638 | 90.066 (88.231-91.939) |
| 2011 | 9581 | 100.659 | 95.183 (93.296-97.108) |
| 2012 | 9828 | 101.361 | 96.960 (95.062-98.896) |
| 2013 | 10372 | 99.838 | 103.889 (101.909-105.907) |
| 2014 | 11012 | 99.166 | 111.046 (108.991-113.140) |
| 2015 | 12231 | 99.478 | 122.952 (120.792-125.150) |
| 2016 | 13304 | 100.255 | 132.701 (130.465-134.976) |
| 2017 | 14738 | 101.268 | 145.535 (143.205-147.904) |
| 2018 | 15892 | 102.568 | 154.940 (152.550-157.368) |
| 2019 | 17515 | 102.460 | 170.945 (168.432-173.496) |
| **Narcolepsy (full population)** | | | |
| 2000-2004 | 564 | 557.815 | 1.011 (0.931-1.098) |
| 2005-2009 | 616 | 608.595 | 1.012 (0.935-1.095) |
| 2010-2014 | 695 | 632.154 | 1.099 (1.021-1.184) |
| 2015-2019 | 761 | 645.967 | 1.178 (1.097-1.265) |

**SA Table 4:** Diagnosed prevalence of Obstructive Sleep Apneoa (OSA) and narcolepsy in England from 2000 to 2019

*directly age/sex standardised to Office of National Statistics population figures for England for each year

| **Year** | **Prev-alent cases (n)** | **Study pop-ulation (n)** | **Crude prevalence % (95% CI)** | **Standardised* prevalence % (95% CI)** | **Estimated cases in the population n (95% plausible interval)** |
| --- | --- | --- | --- | --- | --- |
| **Obstructive Sleep Apnoea (adults only)** | | | | | |
| 2000 | 10305 | 8593444 | 0.120 (0.118-0.122) | 0.116 (0.113-0.118) | 43,959 (43,110-44,807) |
| 2001 | 13012 | 8820998 | 0.148 (0.145-0.150) | 0.142 (0.140-0.145) | 54,522 (53,586-55,458) |
| 2002 | 16223 | 8997943 | 0.180 (0.178-0.183) | 0.174 (0.171-0.177) | 67,125 (66,093-68,157) |
| 2003 | 19905 | 9198929 | 0.216 (0.213-0.219) | 0.209 (0.206-0.212) | 81,260 (80,133-82,388) |
| 2004 | 23738 | 9345873 | 0.254 (0.251-0.257) | 0.246 (0.243-0.249) | 96,091 (94,871-97,312) |
| 2005 | 27820 | 9471842 | 0.294 (0.290-0.297) | 0.285 (0.281-0.288) | 112,392 (111,074-113,711) |
| 2006 | 32444 | 9635545 | 0.337 (0.333-0.340) | 0.327 (0.323-0.331) | 130,319 (128,904-131,734) |
| 2007 | 37566 | 9796975 | 0.383 (0.380-0.387) | 0.373 (0.369-0.377) | 150,075 (148,561-151,588) |
| 2008 | 44147 | 9913300 | 0.445 (0.441-0.449) | 0.434 (0.430-0.438) | 176,396 (174,756-178,036) |
| 2009 | 51623 | 10022265 | 0.515 (0.511-0.520) | 0.504 (0.500-0.509) | 206,620 (204,844-208,396) |
| 2010 | 59348 | 10068216 | 0.589 (0.585-0.594) | 0.579 (0.574-0.583) | 239,370 (237,453-241,288) |
| 2011 | 67161 | 10088116 | 0.666 (0.661-0.671) | 0.655 (0.650-0.660) | 273,710 (271,650-275,769) |
| 2012 | 75317 | 10158121 | 0.741 (0.736-0.747) | 0.733 (0.728-0.738) | 308,408 (306,218-310,598) |
| 2013 | 82248 | 9985639 | 0.824 (0.818-0.829) | 0.816 (0.810-0.821) | 345,579 (343,232-347,925) |
| 2014 | 89893 | 9944074 | 0.904 (0.898-0.910) | 0.896 (0.890-0.902) | 382,838 (380,353-385,323) |
| 2015 | 98502 | 9980677 | 0.987 (0.981-0.993) | 0.979 (0.973-0.985) | 422,103 (419,488-424,719) |
| 2016 | 108061 | 10036444 | 1.077 (1.070-1.083) | 1.074 (1.068-1.081) | 467,120 (464,357-469,882) |
| 2017 | 119347 | 10200979 | 1.170 (1.163-1.177) | 1.175 (1.169-1.182) | 514,299 (511,406-517,191) |
| 2018 | 131873 | 10347122 | 1.274 (1.268-1.281) | 1.287 (1.281-1.294) | 566,746 (563,716-569,777) |
| 2019 | 143277 | 10360607 | 1.383 (1.376-1.390) | 1.406 (1.399-1.414) | 622,528 (619,336-625,720) |
| **Narcolepsy (full population)** | | | | | |
| 2000 | 979 | 10649029 | 0.009 (0.009-0.010) | 0.009 (0.008-0.009) | 4,278 (4,009-4,546) |
| 2001 | 1067 | 10935908 | 0.010 (0.009-0.010) | 0.009 (0.009-0.010) | 4,578 (4,303-4,853) |
| 2002 | 1193 | 11157068 | 0.011 (0.010-0.011) | 0.010 (0.010-0.011) | 5,050 (4,763-5,337) |
| 2003 | 1305 | 11413180 | 0.011 (0.011-0.012) | 0.011 (0.010-0.012) | 5,458 (5,161-5,755) |
| 2004 | 1431 | 11605453 | 0.012 (0.012-0.013) | 0.012 (0.011-0.012) | 5,938 (5,629-6,246) |
| 2005 | 1516 | 11777450 | 0.013 (0.012-0.014) | 0.012 (0.012-0.013) | 6,279 (5,962-6,595) |
| 2006 | 1612 | 11992543 | 0.013 (0.013-0.014) | 0.013 (0.012-0.014) | 6,620 (6,297-6,944) |
| 2007 | 1703 | 12208739 | 0.014 (0.013-0.015) | 0.014 (0.013-0.014) | 6,951 (6,620-7,282) |
| 2008 | 1823 | 12375754 | 0.015 (0.014-0.015) | 0.014 (0.014-0.015) | 7,423 (7,081-7,764) |
| 2009 | 1919 | 12533001 | 0.015 (0.015-0.016) | 0.015 (0.014-0.016) | 7,791 (7,442-8,140) |
| 2010 | 2027 | 12606257 | 0.016 (0.015-0.017) | 0.016 (0.015-0.016) | 8,283 (7,922-8,644) |
| 2011 | 2095 | 12668578 | 0.017 (0.016-0.017) | 0.016 (0.016-0.017) | 8,612 (8,242-8,981) |
| 2012 | 2192 | 12784860 | 0.017 (0.016-0.018) | 0.017 (0.016-0.018) | 9,017 (8,639-9,395) |
| 2013 | 2211 | 12605317 | 0.018 (0.017-0.018) | 0.017 (0.017-0.018) | 9,312 (8,924-9,701) |
| 2014 | 2287 | 12585025 | 0.018 (0.017-0.019) | 0.018 (0.017-0.019) | 9,753 (9,353-10,153) |
| 2015 | 2356 | 12646881 | 0.019 (0.018-0.019) | 0.018 (0.018-0.019) | 10,102 (9,693-10,510) |
| 2016 | 2406 | 12736996 | 0.019 (0.018-0.020) | 0.019 (0.018-0.020) | 10,364 (9,949-10,779) |
| 2017 | 2470 | 12959549 | 0.019 (0.018-0.020) | 0.019 (0.018-0.020) | 10,531 (10,115-10,948) |
| 2018 | 2573 | 13146928 | 0.020 (0.019-0.020) | 0.019 (0.019-0.020) | 10,885 (10,463-11,306) |
| 2019 | 2662 | 13159742 | 0.020 (0.019-0.021) | 0.020 (0.019-0.021) | 11,307 (10,876-11,738) |

**SA Table 5:** Crude incidence rates by year and country in primary care only study populations (obstructive sleep apnoea - OSA)

| **Calendar year** | **Incident cases (n)** | **Person years / 100 000** | **Crude incidence rate per 100 000 person years (95% CI)** |
| --- | --- | --- | --- |
| **United Kingdom** | | | |
| 2000 | 2453 | 127.359 | 19.260 (18.513-20.038) |
| 2001 | 3029 | 129.819 | 23.333 (22.516-24.178) |
| 2002 | 3586 | 132.260 | 27.113 (26.240-28.015) |
| 2003 | 4242 | 134.726 | 31.486 (30.553-32.448) |
| 2004 | 4729 | 137.111 | 34.490 (33.521-35.488) |
| 2005 | 5146 | 138.603 | 37.128 (36.127-38.156) |
| 2006 | 5833 | 140.255 | 41.588 (40.535-42.669) |
| 2007 | 6678 | 141.968 | 47.039 (45.924-48.181) |
| 2008 | 8529 | 143.625 | 59.384 (58.137-60.658) |
| 2009 | 9020 | 144.120 | 62.587 (61.308-63.892) |
| 2010 | 9294 | 144.568 | 64.288 (62.994-65.608) |
| 2011 | 9747 | 144.611 | 67.402 (66.077-68.753) |
| 2012 | 10233 | 145.612 | 70.276 (68.927-71.651) |
| 2013 | 10760 | 142.655 | 75.427 (74.015-76.866) |
| 2014 | 11337 | 140.985 | 80.413 (78.946-81.907) |
| 2015 | 12633 | 140.848 | 89.692 (88.142-91.270) |
| 2016 | 13859 | 141.321 | 98.067 (96.448-99.714) |
| 2017 | 15290 | 142.106 | 107.596 (105.904-109.315) |
| 2018 | 16383 | 143.551 | 114.127 (112.393-115.888) |
| 2019 | 18403 | 143.734 | 128.035 (126.199-129.899) |
| **England** | | | |
| 2000 | 2068 | 107.633 | 19.213 (18.403-20.060) |
| 2001 | 2634 | 109.860 | 23.976 (23.078-24.909) |
| 2002 | 3137 | 112.085 | 27.988 (27.025-28.984) |
| 2003 | 3665 | 114.341 | 32.053 (31.032-33.108) |
| 2004 | 4037 | 116.278 | 34.719 (33.664-35.806) |
| 2005 | 4409 | 117.507 | 37.521 (36.430-38.645) |
| 2006 | 4972 | 118.858 | 41.831 (40.685-43.010) |
| 2007 | 5686 | 120.312 | 47.260 (46.048-48.505) |
| 2008 | 7350 | 121.643 | 60.423 (59.057-61.820) |
| 2009 | 7762 | 121.998 | 63.624 (62.224-65.055) |
| 2010 | 7948 | 122.209 | 65.036 (63.622-66.482) |
| 2011 | 8363 | 122.064 | 68.513 (67.061-69.998) |
| 2012 | 8748 | 122.810 | 71.232 (69.755-72.740) |
| 2013 | 9173 | 119.778 | 76.583 (75.032-78.167) |
| 2014 | 9672 | 118.002 | 81.965 (80.347-83.615) |
| 2015 | 10920 | 117.893 | 92.627 (90.906-94.380) |
| 2016 | 11881 | 118.405 | 100.342 (98.554-102.163) |
| 2017 | 13314 | 119.298 | 111.603 (109.723-113.515) |
| 2018 | 14322 | 120.558 | 118.798 (116.868-120.759) |
| 2019 | 16145 | 120.558 | 133.919 (131.869-136.001) |
| **England** | | | |
| 2000-2004 | 15541 | 560.198 | 27.742 (27.309-28.182) |
| 2005-2009 | 30179 | 600.318 | 50.272 (49.708-50.842) |
| 2010-2014 | 43904 | 604.863 | 72.585 (71.909-73.267) |
| 2015-2019 | 66582 | 596.711 | 111.582 (110.737-112.432) |
| **Wales** | | | |
| 2000-2004 | 921 | 34.324 | 26.832 (25.154-28.623) |
| 2005-2009 | 1690 | 38.271 | 44.159 (42.103-46.316) |
| 2010-2014 | 2581 | 40.647 | 63.498 (61.095-65.996) |
| 2015-2019 | 3914 | 40.116 | 97.567 (94.558-100.672) |
| **Scotland** | | | |
| 2000-2004 | 1388 | 54.525 | 25.456 (24.152-26.831) |
| 2005-2009 | 2729 | 57.039 | 47.845 (46.083-49.674) |
| 2010-2014 | 3935 | 59.420 | 66.223 (64.186-68.325) |
| 2015-2019 | 4617 | 60.950 | 75.750 (73.596-77.967) |
| **Northern Ireland** | | | |
| 2000-2004 | 189 | 12.228 | 15.456 (13.403-17.825) |
| 2005-2009 | 608 | 12.943 | 46.974 (43.385-50.860) |
| 2010-2014 | 951 | 13.500 | 70.444 (66.106-75.066) |
| 2015-2019 | 1455 | 13.782 | 105.569 (100.281-111.135) |

**SA Table 6:** Crude incidence rates by year and country in primary care only study populations (narcolepsy)

| **Calendar year** | **Incident cases (n)** | **Person years / 100 000** | **Crude incidence rate per 100 000 person years (95% CI)** |
| --- | --- | --- | --- |
| **United Kingdom** | | | |
| 2000-2004 | 683 | 816.888 | 0.836 (0.776-0.901) |
| 2005-2009 | 727 | 878.156 | 0.828 (0.770-0.890) |
| 2010-2014 | 752 | 900.179 | 0.835 (0.778-0.897) |
| 2015-2019 | 708 | 903.324 | 0.784 (0.728-0.844) |
| **England** | | | |
| 2000-2004 | 584 | 691.087 | 0.845 (0.779-0.916) |
| 2005-2009 | 618 | 743.677 | 0.831 (0.768-0.899) |
| 2010-2014 | 643 | 758.543 | 0.848 (0.785-0.916) |
| 2015-2019 | 624 | 759.485 | 0.822 (0.760-0.889) |
| **Wales** | | | |
| 2000-2004 | 30 | 42.469 | 0.706 (0.494-1.010) |
| 2005-2009 | 38 | 47.292 | 0.804 (0.585-1.104) |
| 2010-2014 | 44 | 50.509 | 0.871 (0.648-1.171) |
| 2015-2019 | 33 | 50.238 | 0.657 (0.467-0.924) |
| **Scotland** | | | |
| 2000-2004 | 54 | 67.222 | 0.803 (0.615-1.049) |
| 2005-2009 | 51 | 70.294 | 0.726 (0.551-0.955) |
| 2010-2014 | 53 | 73.503 | 0.721 (0.551-0.944) |
| 2015-2019 | 46 | 75.548 | 0.609 (0.456-0.813) |
| **Northern Ireland** | | | |
| 2000-2004 | 15 | 16.110 | 0.931 (0.561-1.544) |
| 2005-2009 | 20 | 16.892 | 1.184 (0.764-1.835) |
| 2010-2014 | 12 | 17.624 | 0.681 (0.387-1.199) |
| 2015-2019 | 5 | 18.053 | 0.277 (0.115-0.665) |

**SA Table 7:** Crude and standardised prevalence by year and country in primary care only study populations (obstructive sleep apnoea - OSA)

| **Year** | **Prevalent cases (n)** | **Study population (n)** | **Crude prevalence % (95% CI)** | **Standardised prevalence %**  **(95% CI)** | **Estimated cases in the population n (95% plausible interval)** |
| --- | --- | --- | --- | --- | --- |
| **United Kingdom** | | | | | |
| 2000 | 11481 | 12638050 | 0.091 (0.089-0.093) | 0.091 (0.089-0.093) | 41,316 (40,561-42,071) |
| 2001 | 14311 | 12931397 | 0.111 (0.109-0.112) | 0.111 (0.109-0.112) | 50,638 (49,809-51,466) |
| 2002 | 17756 | 13162026 | 0.135 (0.133-0.137) | 0.135 (0.133-0.137) | 62,120 (61,208-63,033) |
| 2003 | 21819 | 13413640 | 0.163 (0.161-0.165) | 0.163 (0.161-0.165) | 75,400 (74,401-76,399) |
| 2004 | 26488 | 13625048 | 0.194 (0.192-0.197) | 0.194 (0.192-0.197) | 90,767 (89,677-91,858) |
| 2005 | 31291 | 13793276 | 0.227 (0.224-0.229) | 0.227 (0.224-0.229) | 106,992 (105,809-108,174) |
| 2006 | 36647 | 13974812 | 0.262 (0.260-0.265) | 0.262 (0.260-0.265) | 124,802 (123,528-126,076) |
| 2007 | 42398 | 14162557 | 0.299 (0.297-0.302) | 0.299 (0.297-0.302) | 143,825 (142,460-145,190) |
| 2008 | 49640 | 14299943 | 0.347 (0.344-0.350) | 0.347 (0.344-0.350) | 168,358 (166,882-169,833) |
| 2009 | 57810 | 14394476 | 0.402 (0.398-0.405) | 0.402 (0.398-0.405) | 196,428 (194,833-198,023) |
| 2010 | 66008 | 14445072 | 0.457 (0.453-0.460) | 0.457 (0.453-0.460) | 225,606 (223,893-227,319) |
| 2011 | 73949 | 14459113 | 0.511 (0.508-0.515) | 0.511 (0.508-0.515) | 254,894 (253,066-256,722) |
| 2012 | 82654 | 14552674 | 0.568 (0.564-0.572) | 0.568 (0.564-0.572) | 285,010 (283,078-286,943) |
| 2013 | 90298 | 14221769 | 0.635 (0.631-0.639) | 0.635 (0.631-0.639) | 320,652 (318,573-322,730) |
| 2014 | 98766 | 14105323 | 0.700 (0.696-0.705) | 0.700 (0.696-0.705) | 356,471 (354,263-358,679) |
| 2015 | 107817 | 14098914 | 0.765 (0.760-0.769) | 0.765 (0.760-0.769) | 392,600 (390,274-394,927) |
| 2016 | 117851 | 14114587 | 0.835 (0.830-0.840) | 0.835 (0.830-0.840) | 432,238 (429,789-434,686) |
| 2017 | 129704 | 14267881 | 0.909 (0.904-0.914) | 0.909 (0.904-0.914) | 473,427 (470,872-475,981) |
| 2018 | 143167 | 14431199 | 0.992 (0.987-0.997) | 0.992 (0.987-0.997) | 519,683 (517,016-522,351) |
| 2019 | 155945 | 14480286 | 1.077 (1.072-1.082) | 1.077 (1.072-1.082) | 567,265 (564,477-570,053) |
| **England** | | | | | |
| 2000 | 9662 | 10680146 | 0.090 (0.089-0.093) | 0.091 (0.089-0.092) | 34,447 (33,761-35,133) |
| 2001 | 12068 | 10944400 | 0.110 (0.109-0.112) | 0.110 (0.108-0.112) | 42,282 (41,529-43,036) |
| 2002 | 15071 | 11155898 | 0.135 (0.133-0.137) | 0.135 (0.133-0.137) | 52,176 (51,344-53,008) |
| 2003 | 18575 | 11389635 | 0.163 (0.161-0.165) | 0.163 (0.161-0.166) | 63,450 (62,539-64,361) |
| 2004 | 22518 | 11555450 | 0.195 (0.192-0.197) | 0.195 (0.193-0.198) | 76,372 (75,377-77,368) |
| 2005 | 26583 | 11695614 | 0.227 (0.224-0.229) | 0.228 (0.225-0.231) | 90,056 (88,976-91,136) |
| 2006 | 31154 | 11845333 | 0.263 (0.260-0.265) | 0.264 (0.261-0.267) | 105,190 (104,025-106,355) |
| 2007 | 35925 | 12002535 | 0.299 (0.297-0.302) | 0.300 (0.297-0.304) | 120,870 (119,624-122,116) |
| 2008 | 42198 | 12116052 | 0.348 (0.344-0.350) | 0.350 (0.346-0.353) | 142,061 (140,711-143,412) |
| 2009 | 49208 | 12190743 | 0.404 (0.398-0.405) | 0.406 (0.402-0.409) | 166,142 (164,680-167,604) |
| 2010 | 56116 | 12214594 | 0.459 (0.453-0.460) | 0.462 (0.458-0.466) | 191,002 (189,429-192,575) |
| 2011 | 62833 | 12207407 | 0.515 (0.508-0.515) | 0.518 (0.514-0.522) | 216,154 (214,473-217,836) |
| 2012 | 70296 | 12280638 | 0.572 (0.564-0.572) | 0.576 (0.572-0.580) | 242,219 (240,439-244,000) |
| 2013 | 76524 | 11935135 | 0.641 (0.631-0.639) | 0.645 (0.640-0.650) | 273,234 (271,310-275,158) |
| 2014 | 83598 | 11806362 | 0.708 (0.696-0.705) | 0.712 (0.708-0.717) | 304,346 (302,297-306,396) |
| 2015 | 91362 | 11803821 | 0.774 (0.760-0.769) | 0.779 (0.774-0.784) | 335,657 (333,496-337,818) |
| 2016 | 100054 | 11829250 | 0.846 (0.830-0.840) | 0.851 (0.846-0.857) | 370,166 (367,891-372,442) |
| 2017 | 110341 | 11983845 | 0.921 (0.904-0.914) | 0.927 (0.922-0.933) | 405,694 (403,321-408,068) |
| 2018 | 122087 | 12124239 | 1.007 (0.987-0.997) | 1.015 (1.009-1.020) | 446,657 (444,174-449,139) |
| 2019 | 133036 | 12151605 | 1.095 (1.072-1.082) | 1.104 (1.098-1.110) | 488,662 (486,062-491,262) |
| **Wales** | | | | | |
| 2000 | 631 | 651294 | 0.097 (0.089-0.093) | 0.094 (0.087-0.101) | 2,109 (1,945-2,274) |
| 2001 | 766 | 667724 | 0.115 (0.109-0.112) | 0.111 (0.103-0.119) | 2,498 (2,321-2,675) |
| 2002 | 922 | 682012 | 0.135 (0.133-0.137) | 0.131 (0.122-0.139) | 2,963 (2,771-3,154) |
| 2003 | 1149 | 691811 | 0.166 (0.161-0.165) | 0.161 (0.151-0.170) | 3,671 (3,458-3,883) |
| 2004 | 1452 | 715018 | 0.203 (0.192-0.197) | 0.196 (0.186-0.206) | 4,526 (4,292-4,759) |
| 2005 | 1727 | 732836 | 0.236 (0.224-0.229) | 0.227 (0.216-0.237) | 5,263 (5,015-5,512) |
| 2006 | 1990 | 748841 | 0.266 (0.260-0.265) | 0.255 (0.244-0.266) | 5,971 (5,708-6,234) |
| 2007 | 2353 | 763912 | 0.308 (0.297-0.302) | 0.295 (0.283-0.307) | 6,974 (6,692-7,256) |
| 2008 | 2670 | 777488 | 0.343 (0.344-0.350) | 0.329 (0.316-0.341) | 7,849 (7,551-8,147) |
| 2009 | 3041 | 785195 | 0.387 (0.398-0.405) | 0.370 (0.356-0.383) | 8,881 (8,565-9,196) |
| 2010 | 3495 | 799792 | 0.437 (0.453-0.460) | 0.417 (0.403-0.431) | 10,077 (9,743-10,411) |
| 2011 | 3877 | 808406 | 0.480 (0.508-0.515) | 0.457 (0.443-0.471) | 11,110 (10,761-11,460) |
| 2012 | 4311 | 814743 | 0.529 (0.564-0.572) | 0.502 (0.487-0.517) | 12,265 (11,900-12,631) |
| 2013 | 4836 | 818329 | 0.591 (0.631-0.639) | 0.561 (0.545-0.577) | 13,763 (13,376-14,150) |
| 2014 | 5338 | 821261 | 0.650 (0.696-0.705) | 0.617 (0.601-0.634) | 15,201 (14,794-15,607) |
| 2015 | 5683 | 800490 | 0.710 (0.760-0.769) | 0.675 (0.657-0.692) | 16,677 (16,244-17,109) |
| 2016 | 6089 | 788908 | 0.772 (0.830-0.840) | 0.732 (0.714-0.750) | 18,189 (17,734-18,645) |
| 2017 | 6784 | 799511 | 0.849 (0.904-0.914) | 0.802 (0.783-0.821) | 20,024 (19,549-20,499) |
| 2018 | 7554 | 807107 | 0.936 (0.987-0.997) | 0.883 (0.863-0.903) | 22,145 (21,647-22,642) |
| 2019 | 8276 | 812719 | 1.018 (1.072-1.082) | 0.957 (0.937-0.978) | 24,155 (23,637-24,674) |
| **Scotland** | | | | | |
| 2000 | 1044 | 1068164 | 0.098 (0.089-0.093) | 0.098 (0.092-0.103) | 3,858 (3,624-4,092) |
| 2001 | 1306 | 1079795 | 0.121 (0.109-0.112) | 0.120 (0.114-0.127) | 4,777 (4,518-5,036) |
| 2002 | 1565 | 1083317 | 0.144 (0.133-0.137) | 0.143 (0.136-0.150) | 5,689 (5,408-5,971) |
| 2003 | 1865 | 1087398 | 0.172 (0.161-0.165) | 0.169 (0.162-0.177) | 6,757 (6,450-7,063) |
| 2004 | 2235 | 1104312 | 0.202 (0.192-0.197) | 0.200 (0.191-0.208) | 8,017 (7,685-8,349) |
| 2005 | 2617 | 1112463 | 0.235 (0.224-0.229) | 0.232 (0.223-0.240) | 9,381 (9,022-9,740) |
| 2006 | 3062 | 1123488 | 0.273 (0.260-0.265) | 0.268 (0.259-0.278) | 10,939 (10,552-11,325) |
| 2007 | 3552 | 1135077 | 0.313 (0.297-0.302) | 0.308 (0.298-0.318) | 12,664 (12,248-13,079) |
| 2008 | 4088 | 1149682 | 0.356 (0.344-0.350) | 0.348 (0.338-0.359) | 14,452 (14,010-14,894) |
| 2009 | 4714 | 1158018 | 0.407 (0.398-0.405) | 0.398 (0.387-0.409) | 16,627 (16,154-17,100) |
| 2010 | 5415 | 1167556 | 0.464 (0.453-0.460) | 0.453 (0.441-0.465) | 19,088 (18,581-19,594) |
| 2011 | 6098 | 1176601 | 0.518 (0.508-0.515) | 0.505 (0.492-0.517) | 21,486 (20,948-22,023) |
| 2012 | 6748 | 1188390 | 0.568 (0.564-0.572) | 0.553 (0.539-0.566) | 23,624 (23,063-24,185) |
| 2013 | 7446 | 1197003 | 0.622 (0.631-0.639) | 0.606 (0.592-0.620) | 26,012 (25,424-26,600) |
| 2014 | 8131 | 1203149 | 0.676 (0.696-0.705) | 0.659 (0.645-0.673) | 28,440 (27,825-29,055) |
| 2015 | 8845 | 1216609 | 0.727 (0.760-0.769) | 0.709 (0.695-0.724) | 30,796 (30,158-31,434) |
| 2016 | 9607 | 1226582 | 0.783 (0.830-0.840) | 0.762 (0.747-0.777) | 33,308 (32,646-33,970) |
| 2017 | 10175 | 1210711 | 0.840 (0.904-0.914) | 0.815 (0.799-0.831) | 35,818 (35,126-36,509) |
| 2018 | 10912 | 1222661 | 0.892 (0.987-0.997) | 0.864 (0.848-0.880) | 38,103 (37,392-38,813) |
| 2019 | 11745 | 1235059 | 0.951 (1.072-1.082) | 0.918 (0.901-0.934) | 40,687 (39,956-41,418) |
| **Northern Ireland** | | | | | |
| 2000 | 144 | 238446 | 0.060 (0.089-0.093) | 0.064 (0.053-0.074) | 781 (653-908) |
| 2001 | 171 | 239478 | 0.071 (0.109-0.112) | 0.075 (0.064-0.086) | 925 (786-1,064) |
| 2002 | 198 | 240799 | 0.082 (0.133-0.137) | 0.086 (0.074-0.098) | 1,080 (929-1,230) |
| 2003 | 230 | 244796 | 0.094 (0.161-0.165) | 0.098 (0.085-0.110) | 1,236 (1,076-1,396) |
| 2004 | 283 | 250268 | 0.113 (0.192-0.197) | 0.117 (0.104-0.131) | 1,501 (1,326-1,676) |
| 2005 | 364 | 252363 | 0.144 (0.224-0.229) | 0.149 (0.133-0.164) | 1,926 (1,728-2,124) |
| 2006 | 441 | 257150 | 0.171 (0.260-0.265) | 0.177 (0.160-0.193) | 2,321 (2,104-2,537) |
| 2007 | 568 | 261033 | 0.218 (0.297-0.302) | 0.223 (0.205-0.242) | 2,974 (2,729-3,218) |
| 2008 | 684 | 256721 | 0.266 (0.344-0.350) | 0.272 (0.252-0.293) | 3,672 (3,397-3,947) |
| 2009 | 847 | 260520 | 0.325 (0.398-0.405) | 0.331 (0.308-0.353) | 4,509 (4,206-4,812) |
| 2010 | 982 | 263130 | 0.373 (0.453-0.460) | 0.378 (0.355-0.402) | 5,200 (4,876-5,525) |
| 2011 | 1141 | 266699 | 0.428 (0.508-0.515) | 0.432 (0.407-0.456) | 5,969 (5,624-6,315) |
| 2012 | 1299 | 268903 | 0.483 (0.564-0.572) | 0.484 (0.458-0.510) | 6,735 (6,370-7,100) |
| 2013 | 1492 | 271302 | 0.550 (0.631-0.639) | 0.549 (0.521-0.577) | 7,671 (7,284-8,059) |
| 2014 | 1699 | 274551 | 0.619 (0.696-0.705) | 0.617 (0.588-0.646) | 8,684 (8,273-9,095) |
| 2015 | 1927 | 277994 | 0.693 (0.760-0.769) | 0.690 (0.659-0.720) | 9,776 (9,342-10,210) |
| 2016 | 2101 | 269847 | 0.779 (0.830-0.840) | 0.776 (0.743-0.809) | 11,072 (10,602-11,542) |
| 2017 | 2404 | 273814 | 0.878 (0.904-0.914) | 0.870 (0.836-0.905) | 12,487 (11,991-12,982) |
| 2018 | 2614 | 277192 | 0.943 (0.987-0.997) | 0.931 (0.896-0.967) | 13,443 (12,932-13,954) |
| 2019 | 2888 | 280903 | 1.028 (1.072-1.082) | 1.010 (0.974-1.047) | 14,677 (14,146-15,208) |

**SA Table 8:** Crude and standardised prevalence by year and country in primary care only study populations (narcolepsy)

|  | **Prevalent cases (n)** | **Study population (n)** | **Crude prevalence % (95% CI)** | **Standardised prevalence %**  **(95% CI)** | **Estimated cases in the population n (95% plausible interval)** |
| --- | --- | --- | --- | --- | --- |
| **United Kingdom** | | | | | |
| 2000 | 1319 | 15690167 | 0.008 (0.008-0.009) | 0.008 (0.008-0.009) | 4,950 (4,683-5,217) |
| 2001 | 1442 | 16055263 | 0.009 (0.009-0.009) | 0.009 (0.009-0.009) | 5,309 (5,035-5,583) |
| 2002 | 1603 | 16333977 | 0.010 (0.009-0.010) | 0.010 (0.009-0.010) | 5,826 (5,541-6,111) |
| 2003 | 1746 | 16648376 | 0.010 (0.010-0.011) | 0.010 (0.010-0.011) | 6,254 (5,961-6,548) |
| 2004 | 1904 | 16916100 | 0.011 (0.011-0.012) | 0.011 (0.011-0.012) | 6,748 (6,445-7,051) |
| 2005 | 2031 | 17136120 | 0.012 (0.011-0.012) | 0.012 (0.011-0.012) | 7,160 (6,849-7,472) |
| 2006 | 2151 | 17363686 | 0.012 (0.012-0.013) | 0.012 (0.012-0.013) | 7,535 (7,217-7,854) |
| 2007 | 2288 | 17606086 | 0.013 (0.012-0.014) | 0.013 (0.012-0.014) | 7,969 (7,642-8,295) |
| 2008 | 2431 | 17796617 | 0.014 (0.013-0.014) | 0.014 (0.013-0.014) | 8,445 (8,109-8,781) |
| 2009 | 2500 | 17941081 | 0.014 (0.013-0.014) | 0.014 (0.013-0.014) | 8,676 (8,336-9,016) |
| 2010 | 2613 | 18019128 | 0.015 (0.014-0.015) | 0.015 (0.014-0.015) | 9,101 (8,752-9,450) |
| 2011 | 2674 | 18079655 | 0.015 (0.014-0.015) | 0.015 (0.014-0.015) | 9,360 (9,005-9,715) |
| 2012 | 2778 | 18230035 | 0.015 (0.015-0.016) | 0.015 (0.015-0.016) | 9,708 (9,347-10,069) |
| 2013 | 2802 | 17889030 | 0.016 (0.015-0.016) | 0.016 (0.015-0.016) | 10,041 (9,669-10,413) |
| 2014 | 2883 | 17797535 | 0.016 (0.016-0.017) | 0.016 (0.016-0.017) | 10,464 (10,082-10,846) |
| 2015 | 2909 | 17813561 | 0.016 (0.016-0.017) | 0.016 (0.016-0.017) | 10,633 (10,246-11,019) |
| 2016 | 2942 | 17860814 | 0.016 (0.016-0.017) | 0.016 (0.016-0.017) | 10,813 (10,423-11,204) |
| 2017 | 2990 | 18075482 | 0.017 (0.016-0.017) | 0.017 (0.016-0.017) | 10,924 (10,533-11,316) |
| 2018 | 3102 | 18284633 | 0.017 (0.016-0.018) | 0.017 (0.016-0.018) | 11,271 (10,874-11,667) |
| 2019 | 3144 | 18342324 | 0.017 (0.017-0.018) | 0.017 (0.017-0.018) | 11,449 (11,049-11,850) |
| **England** | | | | | |
| 2000 | 1119 | 13237155 | 0.008 (0.008-0.009) | 0.008 (0.008-0.009) | 4,158 (3,914-4,402) |
| 2001 | 1219 | 13566851 | 0.009 (0.009-0.009) | 0.009 (0.008-0.009) | 4,441 (4,192-4,690) |
| 2002 | 1362 | 13823413 | 0.010 (0.009-0.010) | 0.010 (0.009-0.010) | 4,894 (4,634-5,153) |
| 2003 | 1487 | 14116626 | 0.011 (0.010-0.011) | 0.011 (0.010-0.011) | 5,260 (4,993-5,527) |
| 2004 | 1621 | 14329760 | 0.011 (0.011-0.012) | 0.011 (0.011-0.012) | 5,680 (5,404-5,957) |
| 2005 | 1717 | 14517222 | 0.012 (0.011-0.012) | 0.012 (0.011-0.012) | 5,991 (5,708-6,274) |
| 2006 | 1817 | 14707625 | 0.012 (0.012-0.013) | 0.012 (0.012-0.013) | 6,303 (6,013-6,592) |
| 2007 | 1925 | 14914190 | 0.013 (0.012-0.014) | 0.013 (0.012-0.013) | 6,640 (6,343-6,936) |
| 2008 | 2052 | 15073902 | 0.014 (0.013-0.014) | 0.014 (0.013-0.014) | 7,063 (6,757-7,369) |
| 2009 | 2105 | 15192012 | 0.014 (0.013-0.014) | 0.014 (0.013-0.014) | 7,243 (6,933-7,552) |
| 2010 | 2205 | 15237378 | 0.014 (0.014-0.015) | 0.014 (0.014-0.015) | 7,631 (7,312-7,949) |
| 2011 | 2252 | 15269404 | 0.015 (0.014-0.015) | 0.015 (0.014-0.015) | 7,845 (7,521-8,169) |
| 2012 | 2337 | 15393035 | 0.015 (0.015-0.016) | 0.015 (0.015-0.016) | 8,136 (7,807-8,466) |
| 2013 | 2348 | 15031848 | 0.016 (0.015-0.016) | 0.016 (0.015-0.016) | 8,434 (8,093-8,775) |
| 2014 | 2406 | 14923577 | 0.016 (0.016-0.017) | 0.016 (0.016-0.017) | 8,782 (8,431-9,132) |
| 2015 | 2439 | 14943195 | 0.016 (0.016-0.017) | 0.016 (0.016-0.017) | 8,968 (8,612-9,324) |
| 2016 | 2474 | 15002301 | 0.016 (0.016-0.017) | 0.017 (0.016-0.017) | 9,144 (8,784-9,504) |
| 2017 | 2520 | 15219470 | 0.017 (0.016-0.017) | 0.017 (0.016-0.017) | 9,241 (8,880-9,602) |
| 2018 | 2613 | 15402023 | 0.017 (0.016-0.018) | 0.017 (0.016-0.018) | 9,530 (9,164-9,895) |
| 2019 | 2654 | 15434759 | 0.017 (0.017-0.018) | 0.017 (0.017-0.018) | 9,713 (9,343-10,082) |
| **Wales** | | | | | |
| 2000 | 61 | 811504 | 0.008 (0.008-0.009) | 0.007 (0.005-0.009) | 212 (159-265) |
| 2001 | 67 | 831795 | 0.008 (0.009-0.009) | 0.008 (0.006-0.010) | 226 (172-281) |
| 2002 | 72 | 848153 | 0.008 (0.009-0.010) | 0.008 (0.006-0.010) | 240 (184-295) |
| 2003 | 76 | 859683 | 0.009 (0.010-0.011) | 0.009 (0.007-0.010) | 251 (195-308) |
| 2004 | 84 | 887228 | 0.009 (0.011-0.012) | 0.009 (0.007-0.011) | 271 (213-329) |
| 2005 | 93 | 908639 | 0.010 (0.011-0.012) | 0.010 (0.008-0.012) | 293 (233-352) |
| 2006 | 102 | 928687 | 0.011 (0.012-0.013) | 0.011 (0.009-0.013) | 316 (255-378) |
| 2007 | 111 | 947076 | 0.012 (0.012-0.014) | 0.011 (0.009-0.013) | 341 (277-405) |
| 2008 | 113 | 964451 | 0.012 (0.013-0.014) | 0.011 (0.009-0.014) | 345 (281-409) |
| 2009 | 118 | 974705 | 0.012 (0.013-0.014) | 0.012 (0.010-0.014) | 358 (293-423) |
| 2010 | 120 | 992503 | 0.012 (0.014-0.015) | 0.012 (0.010-0.014) | 357 (293-421) |
| 2011 | 126 | 1004425 | 0.013 (0.014-0.015) | 0.012 (0.010-0.014) | 372 (307-437) |
| 2012 | 135 | 1013274 | 0.013 (0.015-0.016) | 0.013 (0.011-0.015) | 397 (330-464) |
| 2013 | 137 | 1019251 | 0.013 (0.015-0.016) | 0.013 (0.011-0.015) | 400 (333-467) |
| 2014 | 154 | 1024517 | 0.015 (0.016-0.017) | 0.014 (0.012-0.017) | 447 (376-518) |
| 2015 | 151 | 998908 | 0.015 (0.016-0.017) | 0.015 (0.012-0.017) | 451 (379-524) |
| 2016 | 149 | 985176 | 0.015 (0.016-0.017) | 0.015 (0.012-0.017) | 454 (381-528) |
| 2017 | 151 | 999326 | 0.015 (0.016-0.017) | 0.015 (0.012-0.017) | 454 (382-527) |
| 2018 | 161 | 1009351 | 0.016 (0.016-0.018) | 0.015 (0.013-0.018) | 484 (408-559) |
| 2019 | 159 | 1015962 | 0.016 (0.017-0.018) | 0.015 (0.013-0.018) | 479 (404-554) |
| **Scotland** | | | | | |
| 2000 | 120 | 1324178 | 0.009 (0.008-0.009) | 0.009 (0.007-0.011) | 462 (379-545) |
| 2001 | 135 | 1337916 | 0.010 (0.009-0.009) | 0.010 (0.008-0.012) | 515 (428-602) |
| 2002 | 147 | 1342477 | 0.011 (0.009-0.010) | 0.011 (0.009-0.013) | 559 (468-649) |
| 2003 | 158 | 1347412 | 0.012 (0.010-0.011) | 0.012 (0.010-0.014) | 597 (503-690) |
| 2004 | 171 | 1367629 | 0.013 (0.011-0.012) | 0.013 (0.011-0.014) | 640 (544-737) |
| 2005 | 189 | 1376731 | 0.014 (0.011-0.012) | 0.014 (0.012-0.016) | 708 (607-809) |
| 2006 | 196 | 1389048 | 0.014 (0.012-0.013) | 0.014 (0.012-0.016) | 727 (625-828) |
| 2007 | 210 | 1402461 | 0.015 (0.012-0.014) | 0.015 (0.013-0.017) | 775 (670-880) |
| 2008 | 221 | 1421974 | 0.016 (0.013-0.014) | 0.016 (0.013-0.018) | 808 (701-915) |
| 2009 | 230 | 1433083 | 0.016 (0.013-0.014) | 0.016 (0.014-0.018) | 837 (729-946) |
| 2010 | 238 | 1444595 | 0.016 (0.014-0.015) | 0.016 (0.014-0.018) | 862 (752-971) |
| 2011 | 245 | 1456576 | 0.017 (0.014-0.015) | 0.017 (0.015-0.019) | 884 (773-995) |
| 2012 | 251 | 1471450 | 0.017 (0.015-0.016) | 0.017 (0.015-0.019) | 900 (789-1,012) |
| 2013 | 260 | 1482537 | 0.018 (0.015-0.016) | 0.017 (0.015-0.019) | 926 (813-1,039) |
| 2014 | 268 | 1489930 | 0.018 (0.016-0.017) | 0.018 (0.016-0.020) | 953 (839-1,067) |
| 2015 | 266 | 1507362 | 0.018 (0.016-0.017) | 0.017 (0.015-0.020) | 938 (825-1,051) |
| 2016 | 270 | 1519442 | 0.018 (0.016-0.017) | 0.018 (0.015-0.020) | 946 (833-1,059) |
| 2017 | 269 | 1497834 | 0.018 (0.016-0.017) | 0.018 (0.015-0.020) | 955 (840-1,069) |
| 2018 | 278 | 1510716 | 0.018 (0.016-0.018) | 0.018 (0.016-0.020) | 979 (863-1,094) |
| 2019 | 283 | 1524757 | 0.019 (0.017-0.018) | 0.018 (0.016-0.020) | 990 (874-1,105) |
| **Northern Ireland** | | | | | |
| 2000 | 19 | 317330 | 0.006 (0.008-0.009) | 0.007 (0.004-0.010) | 116 (64-169) |
| 2001 | 21 | 318701 | 0.007 (0.009-0.009) | 0.007 (0.004-0.011) | 126 (72-180) |
| 2002 | 22 | 319934 | 0.007 (0.009-0.010) | 0.008 (0.004-0.011) | 132 (76-187) |
| 2003 | 25 | 324655 | 0.008 (0.010-0.011) | 0.008 (0.005-0.012) | 145 (88-201) |
| 2004 | 28 | 331483 | 0.008 (0.011-0.012) | 0.009 (0.006-0.013) | 158 (99-217) |
| 2005 | 32 | 333528 | 0.010 (0.011-0.012) | 0.010 (0.007-0.014) | 181 (118-244) |
| 2006 | 36 | 338326 | 0.011 (0.012-0.013) | 0.012 (0.008-0.015) | 202 (136-268) |
| 2007 | 42 | 342359 | 0.012 (0.012-0.014) | 0.013 (0.009-0.017) | 235 (163-306) |
| 2008 | 45 | 336290 | 0.013 (0.013-0.014) | 0.014 (0.010-0.019) | 255 (180-330) |
| 2009 | 47 | 341281 | 0.014 (0.013-0.014) | 0.015 (0.010-0.019) | 264 (188-339) |
| 2010 | 50 | 344652 | 0.015 (0.014-0.015) | 0.015 (0.011-0.020) | 279 (201-356) |
| 2011 | 51 | 349250 | 0.015 (0.014-0.015) | 0.015 (0.011-0.020) | 280 (203-357) |
| 2012 | 55 | 352276 | 0.016 (0.015-0.016) | 0.016 (0.012-0.021) | 299 (220-378) |
| 2013 | 57 | 355394 | 0.016 (0.015-0.016) | 0.017 (0.012-0.021) | 305 (226-385) |
| 2014 | 55 | 359511 | 0.015 (0.016-0.017) | 0.016 (0.012-0.020) | 292 (215-369) |
| 2015 | 53 | 364096 | 0.015 (0.016-0.017) | 0.015 (0.011-0.019) | 277 (202-352) |
| 2016 | 49 | 353895 | 0.014 (0.016-0.017) | 0.014 (0.010-0.018) | 265 (191-339) |
| 2017 | 50 | 358852 | 0.014 (0.016-0.017) | 0.014 (0.010-0.018) | 267 (193-341) |
| 2018 | 50 | 362543 | 0.014 (0.016-0.018) | 0.014 (0.010-0.018) | 267 (193-341) |
| 2019 | 48 | 366846 | 0.013 (0.017-0.018) | 0.013 (0.010-0.017) | 254 (182-326) |

**SA Table 6**: 2019 Prevalence Ratios in England using the primary linked algorithm and primary care only algorithms as a sensitivity analysis (OSA)

Caption (SA tables 6-9): Deprivation and urban rural data were not available for the full primary care population

|  | **Primary algorithm**  **(OSA identified in primary care or linked data)** | | | **Sensitivity algorithm**  **(OSA identified in primary care data only)** | | |
| --- | --- | --- | --- | --- | --- | --- |
|  | Unadjusted | Age and sex adjusted | Age, sex and BMI adjusted | Unadjusted | Age and sex adjusted | Age, sex and BMI adjusted |
| **Age group** | | | | | | |
| 18 to < 25 | 0.08 (0.07-0.08) | 0.08 (0.07-0.09) | 0.16 (0.15-0.18) | 0.07 (0.06-0.08) | 0.07 (0.07-0.08) | 0.15 (0.14-0.16) |
| 25 to < 35 | 0.34 (0.33-0.35) | 0.35 (0.34-0.36) | 0.44 (0.43-0.46) | 0.33 (0.32-0.34) | 0.34 (0.33-0.35) | 0.43 (0.42-0.45) |
| 35 to < 45 | 1 | 1 | 1 | 1 | 1 | 1 |
| 45 to < 55 | 2.10 (2.06-2.14) | 2.10 (2.06-2.14) | 1.65 (1.62-1.68) | 2.11 (2.07-2.15) | 2.11 (2.07-2.15) | 1.63 (1.60-1.67) |
| 55 to < 65 | 3.02 (2.96-3.08) | 3.03 (2.98-3.09) | 2.17 (2.13-2.21) | 3.00 (2.94-3.06) | 3.01 (2.96-3.07) | 2.12 (2.08-2.17) |
| 65 to < 75 | 3.13 (3.07-3.19) | 3.20 (3.14-3.26) | 2.36 (2.32-2.41) | 3.04 (2.98-3.10) | 3.12 (3.06-3.18) | 2.27 (2.23-2.31) |
| 75 to < 85 | 2.20 (2.15-2.25) | 2.31 (2.26-2.37) | 2.01 (1.96-2.06) | 2.04 (1.99-2.08) | 2.15 (2.10-2.20) | 1.86 (1.81-1.90) |
| 85 plus | 0.96 (0.92-1.00) | 1.09 (1.04-1.13) | 1.30 (1.25-1.35) | 0.81 (0.77-0.85) | 0.93 (0.89-0.97) | 1.12 (1.07-1.17) |
| **Sex** | | | | | | |
| male | 1 | 1 | 1 | 1 | 1 | 1 |
| female | 0.42 (0.41-0.42) | 0.42 (0.41-0.42) | 0.34 (0.34-0.35) | 0.37 (0.37-0.38) | 0.37 (0.37-0.38) | 0.31 (0.30-0.31) |
| **BMI category** | | | | | | |
| Underweight | 0.54 (0.49-0.59) | 0.81 (0.73-0.89) | . (.-.) | 0.46 (0.41-0.51) | 0.70 (0.63-0.79) | . (.-.) |
| Normal weight | 1 | 1 | . (.-.) | 1 | 1 | . (.-.) |
| Overweight | 3.31 (3.25-3.38) | 2.36 (2.31-2.41) | . (.-.) | 3.58 (3.50-3.66) | 2.53 (2.47-2.59) | . (.-.) |
| Obesity class I | 7.99 (7.83-8.15) | 5.72 (5.60-5.84) | . (.-.) | 8.97 (8.78-9.17) | 6.40 (6.26-6.54) | . (.-.) |
| Obesity class II | 15.00 (14.68-15.32) | 12.08 (11.83-12.35) | . (.-.) | 17.15 (16.77-17.55) | 13.86 (13.54-14.18) | . (.-.) |
| Obesity class III+ | 29.46 (28.85-30.08) | 26.97 (26.42-27.54) | . (.-.) | 33.34 (32.60-34.11) | 30.87 (30.18-31.57) | . (.-.) |
| **Ethnicity** | | | | | | |
| White | 1 | 1 | 1 | 1 | 1 | 1 |
| South Asian | 0.72 (0.70-0.73) | 0.90 (0.88-0.92) | 1.12 (1.10-1.15) | 0.70 (0.68-0.72) | 0.88 (0.86-0.91) | 1.13 (1.10-1.16) |
| Black | 0.87 (0.85-0.90) | 1.02 (0.99-1.05) | 0.95 (0.93-0.98) | 0.81 (0.79-0.84) | 0.94 (0.91-0.97) | 0.89 (0.86-0.92) |
| Other | 0.45 (0.44-0.47) | 0.69 (0.66-0.72) | 0.93 (0.89-0.97) | 0.40 (0.38-0.43) | 0.66 (0.62-0.70) | 0.95 (0.90-1.01) |
| Mixed | 0.65 (0.62-0.68) | 0.98 (0.93-1.03) | 1.07 (1.02-1.13) | 0.65 (0.61-0.69) | 0.97 (0.91-1.03) | 1.08 (1.02-1.15) |
| **Practice size quintile** | | | | | | |
| 1 largest | 1 | 1 | 1 | 1 | 1 | 1 |
| 2 | 1.13 (1.12-1.15) | 1.01 (1.00-1.03) | 0.98 (0.97-1.00) | 1.12 (1.11-1.14) | 1.01 (0.99-1.02) | 0.98 (0.96-0.99) |
| 3 | 1.13 (1.11-1.14) | 1.00 (0.99-1.02) | 0.94 (0.93-0.96) | 1.11 (1.10-1.13) | 1.00 (0.99-1.02) | 0.94 (0.93-0.96) |
| 4 | 1.15 (1.13-1.17) | 1.02 (1.00-1.03) | 0.94 (0.92-0.95) | 1.14 (1.12-1.16) | 1.02 (1.00-1.03) | 0.94 (0.92-0.95) |
| 5 smallest | 1.10 (1.08-1.12) | 0.97 (0.95-0.99) | 0.87 (0.85-0.88) | 1.08 (1.06-1.11) | 0.96 (0.94-0.98) | 0.86 (0.84-0.88) |

**SA Table 7**: 2019 Incidence Rate Ratios in England using the primary linked algorithm and primary care only algorithms as a sensitivity analysis (OSA)

|  | **Primary algorithm**  **(OSA identified in primary care or linked data)** | | | **Sensitivity algorithm**  **(OSA identified in primary care data only)** | | |
| --- | --- | --- | --- | --- | --- | --- |
|  | Unadjusted | Age and sex adjusted | Age, sex and BMI adjusted | Unadjusted | Age and sex adjusted | Age, sex and BMI adjusted |
| **Age group** | | | | | | |
| 18 to < 25 | 0.20 (0.18-0.22) | 0.20 (0.18-0.23) | 0.36 (0.32-0.42) | 0.11 (0.10-0.12) | 0.11 (0.10-0.13) | 0.20 (0.17-0.23) |
| 25 to < 35 | 0.50 (0.47-0.53) | 0.50 (0.47-0.53) | 0.60 (0.56-0.64) | 0.34 (0.31-0.36) | 0.35 (0.33-0.37) | 0.44 (0.41-0.47) |
| 35 to < 45 | 1 | 1 | 1 | 1 | 1 | 1 |
| 45 to < 55 | 1.56 (1.49-1.64) | 1.57 (1.49-1.64) | 1.26 (1.20-1.32) | 1.93 (1.84-2.03) | 1.91 (1.82-2.00) | 1.48 (1.41-1.55) |
| 55 to < 65 | 1.88 (1.79-1.97) | 1.89 (1.80-1.98) | 1.41 (1.34-1.48) | 2.26 (2.15-2.37) | 2.25 (2.14-2.36) | 1.61 (1.53-1.69) |
| 65 to < 75 | 1.64 (1.56-1.73) | 1.67 (1.58-1.76) | 1.29 (1.22-1.36) | 1.67 (1.58-1.76) | 1.66 (1.58-1.76) | 1.22 (1.16-1.30) |
| 75 to < 85 | 1.17 (1.10-1.25) | 1.22 (1.14-1.30) | 1.09 (1.02-1.16) | 0.75 (0.70-0.81) | 0.76 (0.71-0.82) | 0.68 (0.63-0.73) |
| 85 plus | 0.49 (0.43-0.56) | 0.53 (0.47-0.61) | 0.65 (0.56-0.74) | 0.12 (0.10-0.14) | 0.13 (0.11-0.16) | 0.18 (0.15-0.21) |
| **Sex** | | | | | | |
| male | 1 | 1 | 1 | 1 | 1 | 1 |
| female | 0.55 (0.54-0.57) | 0.56 (0.54-0.57) | 0.43 (0.42-0.45) | 0.45 (0.44-0.47) | 0.52 (0.50-0.54) | 0.44 (0.43-0.46) |
| **BMI category** |  |  |  |  |  |  |
| Normal weight | 1 | 1 | . (.-.) | 1 | 1 | . (.-.) |
| Overweight | 2.80 (2.64-2.96) | 2.24 (2.11-2.37) | . (.-.) | 3.69 (3.48-3.92) | 2.62 (2.46-2.78) | . (.-.) |
| Obesity class I | 6.60 (6.24-6.99) | 5.39 (5.09-5.70) | . (.-.) | 9.27 (8.73-9.84) | 6.38 (6.01-6.78) | . (.-.) |
| Obesity class II | 13.61 (12.83-14.44) | 12.23 (11.52-12.98) | . (.-.) | 18.01 (16.91-19.18) | 13.05 (12.25-13.91) | . (.-.) |
| Obesity class III+ | 30.10 (28.41-31.89) | 30.00 (28.30-31.80) | . (.-.) | 36.13 (33.97-38.42) | 28.01 (26.32-29.81) | . (.-.) |
| **Ethnicity** | | | | | | |
| White | 1 | 1 | 1 | 1 | 1 | 1 |
| South Asian | 0.85 (0.80-0.90) | 0.95 (0.89-1.01) | 1.16 (1.09-1.23) | 1.02 (0.96-1.10) | 1.16 (1.08-1.24) | 1.42 (1.32-1.52) |
| Black | 0.89 (0.82-0.96) | 0.94 (0.87-1.02) | 0.86 (0.79-0.92) | 0.93 (0.85-1.02) | 0.97 (0.88-1.06) | 0.91 (0.83-1.00) |
| Other | 0.54 (0.48-0.60) | 0.68 (0.61-0.76) | 0.92 (0.82-1.03) | 0.35 (0.30-0.41) | 0.57 (0.49-0.67) | 0.82 (0.70-0.95) |
| Mixed | 0.71 (0.62-0.82) | 0.91 (0.79-1.05) | 0.99 (0.85-1.15) | 0.56 (0.47-0.67) | 0.77 (0.64-0.92) | 0.87 (0.73-1.04) |
| **Practice size quintile** | | | | | | |
| 1 largest | 1 | 1 | 1 | 1 | 1 | 1 |
| 2 | 1.11 (1.07-1.16) | 1.03 (0.99-1.07) | 0.99 (0.95-1.03) | 1.36 (1.31-1.42) | 1.16 (1.12-1.21) | 1.11 (1.06-1.16) |
| 3 | 1.10 (1.06-1.15) | 1.02 (0.98-1.07) | 0.95 (0.91-0.99) | 1.13 (1.08-1.18) | 0.92 (0.88-0.96) | 0.88 (0.84-0.92) |
| 4 | 1.17 (1.11-1.22) | 1.08 (1.03-1.13) | 0.98 (0.93-1.03) | 1.34 (1.27-1.40) | 1.07 (1.02-1.12) | 0.97 (0.92-1.02) |
| 5 smallest | 1.06 (1.00-1.12) | 0.97 (0.91-1.03) | 0.84 (0.79-0.90) | 1.22 (1.15-1.30) | 0.98 (0.92-1.04) | 0.84 (0.79-0.90) |

**SA Table 8**: 2015-2019 Prevalence Ratios in in England using the primary linked algorithm and primary care only algorithms as a sensitivity analysis (narcolepsy)

|  | **Primary algorithm**  **(narcolepsy identified in primary care or linked data)** | | **Sensitivity algorithm**  **(narcolepsy identified in primary care data only)** | |
| --- | --- | --- | --- | --- |
|  | Unadjusted | Age and sex adjusted | Unadjusted | Age and sex adjusted |
| **Age group** | | | | |
| 0 to < 9 | 0.06 (0.04-0.09) | 0.06 (0.04-0.09) | 0.05 (0.03-0.07) | 0.05 (0.03-0.07) |
| 9 to < 18 | 0.28 (0.23-0.34) | 0.28 (0.23-0.34) | 0.28 (0.22-0.34) | 0.28 (0.22-0.34) |
| 18 to < 25 | 0.52 (0.44-0.62) | 0.52 (0.43-0.62) | 0.51 (0.43-0.61) | 0.51 (0.43-0.61) |
| 25 to < 35 | 0.74 (0.65-0.84) | 0.74 (0.65-0.84) | 0.73 (0.64-0.84) | 0.73 (0.64-0.83) |
| 35 to < 45 | 1 | 1 | 1 | 1 |
| 45 to < 55 | 0.97 (0.86-1.10) | 0.97 (0.86-1.10) | 0.95 (0.84-1.08) | 0.95 (0.84-1.08) |
| 55 to < 65 | 0.84 (0.74-0.96) | 0.84 (0.73-0.96) | 0.86 (0.75-0.98) | 0.86 (0.75-0.98) |
| 65 to < 75 | 0.84 (0.72-0.97) | 0.84 (0.72-0.97) | 0.86 (0.75-1.00) | 0.86 (0.74-0.99) |
| 75 to < 85 | 1.07 (0.92-1.26) | 1.06 (0.91-1.25) | 1.16 (0.99-1.35) | 1.15 (0.99-1.34) |
| 85 plus | 1.23 (1.00-1.51) | 1.21 (0.98-1.49) | 1.15 (0.93-1.42) | 1.14 (0.92-1.41) |
| **Sex** | | | | |
| male | 1 | 1 | 1 | 1 |
| female | 1.15 (1.06-1.24) | 1.13 (1.05-1.22) | 1.08 (1.00-1.16) | 1.06 (0.98-1.15) |
| **Ethnicity** | | | | |
| White | 1 | 1 | 1 | 1 |
| South Asian | 0.33 (0.27-0.42) | 0.36 (0.29-0.46) | 0.40 (0.32-0.51) | 0.43 (0.34-0.55) |
| Black | 1.40 (1.20-1.62) | 1.51 (1.30-1.75) | 1.29 (1.08-1.55) | 1.39 (1.16-1.67) |
| Other | 0.31 (0.22-0.46) | 0.34 (0.23-0.49) | 0.30 (0.19-0.48) | 0.32 (0.20-0.52) |
| Mixed | 0.91 (0.69-1.20) | 1.24 (0.94-1.63) | 0.87 (0.62-1.22) | 1.12 (0.80-1.57) |
| **Practice size quintile** | | | | |
| 1 largest | 1 | 1 | 1 | 1 |
| 2 | 1.09 (0.99-1.20) | 1.07 (0.97-1.18) | 1.05 (0.95-1.16) | 1.03 (0.93-1.14) |
| 3 | 1.01 (0.90-1.12) | 0.99 (0.88-1.10) | 0.95 (0.85-1.05) | 0.93 (0.83-1.03) |
| 4 | 0.96 (0.85-1.09) | 0.95 (0.84-1.08) | 0.87 (0.77-0.99) | 0.86 (0.76-0.98) |
| 5 smallest | 0.95 (0.82-1.11) | 0.95 (0.81-1.11) | 0.94 (0.81-1.09) | 0.93 (0.80-1.08) |

**SA Table 9:** 2015-2019 Incidence Rate Ratios in England using the primary linked algorithm and primary care only algorithms as a sensitivity analysis (narcolepsy)

|  | **Primary algorithm**  **(narcolepsy identified in primary care or linked data)** | | **Sensitivity algorithm**  **(narcolepsy identified in primary care data only)** | |
| --- | --- | --- | --- | --- |
|  | Unadjusted | Age and sex adjusted | Unadjusted | Age and sex adjusted |
| **Age group** | | | | |
| 0 to < 9 | 0.31 (0.21-0.46) | 0.31 (0.21-0.46) | 0.35 (0.23-0.52) | 0.35 (0.23-0.52) |
| 9 to < 18 | 0.90 (0.69-1.17) | 0.90 (0.69-1.17) | 0.97 (0.73-1.29) | 0.97 (0.73-1.29) |
| 18 to < 25 | 1.12 (0.86-1.46) | 1.12 (0.86-1.46) | 1.40 (1.07-1.83) | 1.39 (1.07-1.82) |
| 25 to < 35 | 1.04 (0.82-1.31) | 1.04 (0.82-1.31) | 1.04 (0.81-1.34) | 1.04 (0.81-1.34) |
| 35 to < 45 | 1 | 1 | 1 | 1 |
| 45 to < 55 | 0.78 (0.60-1.00) | 0.78 (0.60-1.00) | 0.63 (0.47-0.84) | 0.62 (0.47-0.84) |
| 55 to < 65 | 0.62 (0.47-0.83) | 0.62 (0.47-0.83) | 0.50 (0.36-0.70) | 0.50 (0.36-0.70) |
| 65 to < 75 | 0.49 (0.34-0.69) | 0.48 (0.34-0.68) | 0.35 (0.23-0.54) | 0.35 (0.23-0.53) |
| 75 to < 85 | 0.62 (0.42-0.91) | 0.61 (0.42-0.90) | 0.28 (0.16-0.50) | 0.28 (0.16-0.49) |
| 85 plus | 0.82 (0.50-1.32) | 0.79 (0.49-1.27) | 0.49 (0.26-0.94) | 0.47 (0.25-0.90) |
| **Sex** | | | | |
| male | 1 | 1 | 1 | 1 |
| female | 1.27 (1.10-1.46) | 1.28 (1.11-1.47) | 1.33 (1.14-1.56) | 1.35 (1.15-1.59) |
| **Ethnicity** | | | | |
| White | 1 | 1 | 1 | 1 |
| South Asian | 0.52 (0.36-0.74) | 0.49 (0.34-0.70) | 0.56 (0.36-0.87) | 0.50 (0.32-0.78) |
| Black | 1.75 (1.35-2.28) | 1.67 (1.28-2.17) | 1.96 (1.42-2.71) | 1.77 (1.28-2.45) |
| Other | 0.64 (0.38-1.09) | 0.57 (0.34-0.97) | 0.70 (0.35-1.41) | 0.56 (0.28-1.13) |
| Mixed | 1.44 (0.93-2.22) | 1.44 (0.93-2.23) | 1.99 (1.21-3.29) | 1.79 (1.08-2.96) |
| **Practice size quintile** | | | | |
| 1 largest | 1 | 1 | 1 | 1 |
| 2 | 1.05 (0.88-1.27) | 1.09 (0.91-1.31) | 0.83 (0.67-1.02) | 0.88 (0.71-1.08) |
| 3 | 0.95 (0.77-1.17) | 0.99 (0.80-1.21) | 0.79 (0.63-0.99) | 0.84 (0.67-1.05) |
| 4 | 1.06 (0.85-1.32) | 1.10 (0.88-1.37) | 0.80 (0.62-1.03) | 0.85 (0.66-1.09) |
| 5 smallest | 0.92 (0.69-1.23) | 0.96 (0.72-1.28) | 0.87 (0.64-1.18) | 0.92 (0.68-1.25) |

**SA Table 10:** Age-sex adjusted prevalence ratios and incidence rate ratios of OSA in the full study population and restricted to people with complete case BMI data

| **Characteristic** | **Age-sex adjusted Prevalence Ratio** | | **Age-sex adjusted Incidence Rate Ratio** | |
| --- | --- | --- | --- | --- |
|  | **full study population** | **complete case** | **full study population** | **complete case** |
| **Age group** | | | | |
| 18 to < 25 | 0.08 (0.07-0.09) | 0.12 (0.11-0.13) | 0.20 (0.18-0.23) | 0.27 (0.24-0.31) |
| 25 to < 35 | 0.35 (0.34-0.36) | 0.38 (0.37-0.39) | 0.50 (0.47-0.53) | 0.53 (0.50-0.57) |
| 35 to < 45 | 1 | 1 | 1 | 1 |
| 45 to < 55 | 2.10 (2.06-2.14) | 1.99 (1.96-2.03) | 1.57 (1.49-1.64) | 1.49 (1.42-1.57) |
| 55 to < 65 | 3.03 (2.98-3.09) | 2.83 (2.77-2.88) | 1.89 (1.80-1.98) | 1.77 (1.68-1.86) |
| 65 to < 75 | 3.20 (3.14-3.26) | 2.91 (2.86-2.97) | 1.67 (1.58-1.76) | 1.53 (1.45-1.61) |
| 75 to < 85 | 2.31 (2.26-2.37) | 2.08 (2.04-2.13) | 1.22 (1.14-1.30) | 1.11 (1.03-1.18) |
| 85 plus | 1.09 (1.04-1.13) | 1.00 (0.96-1.04) | 0.53 (0.47-0.61) | 0.50 (0.44-0.57) |
| **Sex** | | | | |
| male | 1 | 1 | 1 | 1 |
| female | 0.42 (0.41-0.42) | 0.40 (0.39-0.40) | 0.56 (0.54-0.57) | 0.53 (0.52-0.55) |
| **Ethnicity** | | | | |
| 0. White | 1 | 1 | 1 | 1 |
| 1. South Asian | 0.90 (0.88-0.92) | 0.89 (0.87-0.91) | 0.95 (0.89-1.01) | 0.93 (0.87-0.99) |
| 2. Black | 1.02 (0.99-1.05) | 1.00 (0.97-1.03) | 0.94 (0.87-1.02) | 0.93 (0.86-1.00) |
| 3. Other | 0.69 (0.66-0.72) | 0.69 (0.66-0.72) | 0.68 (0.61-0.76) | 0.68 (0.61-0.77) |
| 4. Mixed | 0.98 (0.93-1.03) | 0.97 (0.92-1.02) | 0.91 (0.79-1.05) | 0.91 (0.78-1.05) |
| **Area based deprivation** | | | | |
| 1 (lowest) | 1 | 1 | 1 | 1 |
| 2 | 0.98 (0.97-1.00) | 0.98 (0.97-1.00) | 1.02 (0.97-1.08) | 1.03 (0.98-1.09) |
| 3 | 1.04 (1.02-1.06) | 1.04 (1.02-1.06) | 1.06 (1.01-1.12) | 1.07 (1.02-1.13) |
| 4 | 1.13 (1.11-1.15) | 1.13 (1.11-1.15) | 1.19 (1.13-1.25) | 1.20 (1.14-1.27) |
| 5 (highest) | 1.11 (1.09-1.13) | 1.10 (1.08-1.12) | 1.16 (1.11-1.23) | 1.18 (1.12-1.25) |
| **Urban-rural** | | | | |
| urban | 1 | 1 | 1 | 1 |
| rural | 0.93 (0.91-0.94) | 0.93 (0.92-0.95) | 0.87 (0.83-0.90) | 0.87 (0.83-0.90) |
| **Practice size quintile** | | | | |
| 1 largest | 1 | 1 | 1 | 1 |
| 2 | 1.01 (1.00-1.03) | 1.01 (1.00-1.03) | 1.03 (0.99-1.07) | 1.03 (0.99-1.08) |
| 3 | 1.00 (0.99-1.02) | 1.00 (0.99-1.02) | 1.02 (0.98-1.07) | 1.02 (0.98-1.07) |
| 4 | 1.02 (1.00-1.03) | 1.01 (0.99-1.03) | 1.08 (1.03-1.13) | 1.08 (1.03-1.13) |
| 5 smallest | 0.97 (0.95-0.99) | 0.96 (0.94-0.98) | 0.97 (0.91-1.03) | 0.95 (0.90-1.01) |
